# Intensity modulated radiotherapy in early stage Hodgkin lymphoma patients: Is it better than three dimensional conformal radiotherapy?

**DOI:** 10.1186/1748-717X-7-129

**Published:** 2012-08-02

**Authors:** Vitaliana De Sanctis, Chiara Bolzan, Marco D’Arienzo, Stefano Bracci, Alessandro Fanelli, Maria Christina Cox, Maurizio Valeriani, Mattia F Osti, Giuseppe Minniti, Laura Chiacchiararelli, Riccardo Maurizi Enrici

**Affiliations:** 1 Departments of Radiotherapy, Sant’Andrea Hospital, Via di Grottarossa 1035/1039, 00189, Rome, Italy; 2 Departments of Medical Physics, Sant’Andrea Hospital, Via di Grottarossa 1035/1039, 00189, Rome, Italy; 3 Centro Ricerche Casaccia, Istituto Nazionale di Metrologia delle Radiazioni Ionizzanti, ENEA, Via Anguillarese 301, 00123, Rome, Italy; 4 Departments of Hematology, Sant’Andrea Hospital, Via di Grottarossa 1035/1039, 00189, Rome, Italy

**Keywords:** Hodgkin, IMRT, 3D-CRT, NTCP

## Abstract

**Background:**

Cure rate of early Hodgkin Lymphoma are high and avoidance of late toxicities is of paramount importance. This comparative study aims to assess the normal tissue sparing capability of intensity-modulated radiation therapy (IMRT) versus standard three-dimensional conformal radiotherapy (3D-CRT) in terms of dose-volume parameters and normal tissue complication probability (NTCP) for different organs at risk in supradiaphragmatic Hodgkin Lymphoma (HL) patients.

**Methods:**

Ten HL patients were actually treated with 3D-CRT and all treatments were then re-planned with IMRT. Dose-volume parameters for thyroid, oesophagus, heart, coronary arteries, lung, spinal cord and breast were evaluated. Dose-volume histograms generated by TPS were analyzed to predict the NTCP for the considered organs at risk, according to different endpoints.

**Results:**

Regarding dose-volume parameters no statistically significant differences were recorded for heart and origin of coronary arteries. We recorded statistically significant lower V30 with IMRT for oesophagus (6.42 vs 0.33, p = 0.02) and lungs (4.7 vs 0.1 p = 0.014 for the left lung and 2.59 vs 0.1 p = 0.017 for the right lung) and lower V20 for spinal cord (17.8 vs 7.2 p = 0.02). Moreover the maximum dose to the spinal cord was lower with IMRT (30.2 vs 19.9, p <0.001). Higher V10 with IMRT for thyroid (64.8 vs 95, p = 0.0019) and V5 for lungs (30.3 vs 44.8, p = 0.03, for right lung and 28.9 vs 48.1, p = 0.001 for left lung) were found, respectively. Higher V5 and V10 for breasts were found with IMRT (V5: 4.14 vs 20.6, p = 0.018 for left breast and 3.3 vs 17, p = 0.059 for right breast; V10: 2.5 vs 13.6 p = 0.035 for left breast and 1.7 vs 11, p = 0.07 for the right breast.) As for the NTCP, our data point out that IMRT is not always likely to significantly increase the NTCP to OARs.

**Conclusions:**

In HL male patients IMRT seems feasible and accurate while for women HL patients IMRT should be used with caution.

## Background

Based on young age at diagnosis for most Hodgkin’s lymphoma (HL) patients and their long survival rate, avoiding late complications of chemo and/or radiotherapy such as myocardial infarction and second neoplasms is an issue of paramount importance.

Different studies indicate that long-term HL survivors experience treatment-related morbidity that impairs thyroid, pulmonary and cardiovascular function [1]. Previous works have shown that the irradiation of the thyroid region is likely to induce 50% risk of developing hypothyroidism and a 20% risk of developing thyroid nodules [2,3]. Other authors observed late pulmonary and cardiac toxicity when these organs are irradiated at a given dose level [4,5]. In particular, prospective data with long-term follow-up showed that young patients treated with mediastinal radiotherapy for Hodgkin lymphoma experienced cardiac disease and an impaired quality of life [6].

Furthermore, for female HL survivors, breast cancer is the most common second malignancy [7], and several studies have demonstrated that women treated with radiotherapy for Hodgkin's lymphoma (HL) have an elevated risk of developing breast cancer compared with the general population [8,9].

Currently, Involved Field Radiation Therapy (IFRT) is considered the standard of care in early stage HL, mainly delivered with three-dimensional conformal radiation therapy (3D-CRT) at mean dose of 30 Gy. Nevertheless, even with IFRT 3D-CRT large treatment volumes may be necessary to encompass more than 2 involved lymphonodal regions and/or bulky lesion, with an irradiation of a large volume of normal tissue. Regarding the long-term toxicity, a dose reduction to organ at risk can be obtained either by lowering the total delivered dose or by reducing the treatment volume. Recently a randomized study by German Hodgkin Study Group has shown a good local control with 20 Gy in low-risk early stage HL [10]. Regarding the target radiation volume, in the on-going EORTC-GELA H10 trial, a further reduction of the target radiation volume was employed using Involved Nodal Radiation Therapy (INRT) [11].

There is growing dosimetric evidence that highly conformal irradiation modalities may improve critical organs sparing in the treatment of lymphoma involving large mediastinal disease volumes, with clinically relevant consequences [12]. Recently, IMRT was employed in HL patients with the aim of reducing the radiation dose to lung, hearth, thyroid, spinal cord and breast [13-19]. Goodman et al. [13] showed that compared to conventional parallel-opposed plans and conformal radiotherapy plans, IMRT could decrease the dose delivered to the lung by 12% and 14%, meanwhile increasing the PTV coverage. In a recent study the normal tissue sparing capability of IMRT in the treatment of Hodgkin's lymphoma was studied. The authors concluded that the forward planned IMRT technique could be easily used for improving PTV conformity while sparing [18].

In the present study we compared the actually 3D-CRT delivered treatment with a simulated IMRT plan in 10 patients with diagnosis of early stage HL, to report about the feasibility and toxicity of IMRT technique in this setting of patients. The normal tissue complication probability (NTCP), evaluated from dose volume histograms (DVHs), was calculated both for 3D-CRT and IMRT plans with the aim of assessing the organs at risk sparing capability of the two dose delivery technique.

## Methods

### Patients

Ten patients (5 male and 5 female) with diagnosis of supra-diaphragmatic HL were enrolled and selected for a comparative dosimetric evaluation between 3D-CRT and IMRT.

All patients underwent pre-chemotherapy FDG-PET/CT imaging and total body CT with iodine intravenous contrast injection. Chemotherapy was administered in 9 patients according to ABVD protocol while in 1 case COOP-ABV protocol was employed because of the young age of the patient [20,21]. All patients were treated with combined therapy consisting in chemotherapy and 3D-CRT according to Involved Field Radiation Therapy (IFRT) volume with a standard fractionation scheme of 2 Gy × 15 fractions. All patients actually treated with 3D-CRT were replanned with IMRT (5 fields techniques) as if the patients should be really treated and dosimetric outcomes of the IMRT treatment plans were compared with standard 3D-CRT plans. Toxicities were recorded and graded according to RTOG criteria [22]. Patients and disease characteristics are shown in Table 1.

**Table 1:** Patients, disease and treatment planning characteristics

**Patient**	**Age**	**Gender**	**Disease stage**	**RT Dose**	**PTV Volume**	**Fields**	
					**(cm**^**3**^**)**	**3D-CRT**	**IMRT**
1	21	F	IIB Bulky	30 Gy	831	2	5
2	21	F	IIA	30 Gy	323	4	5
3	53	F	IIA	30 Gy	553	5	5
4	43	M	IIA	30 Gy	841	5	5
5	13	M	IIA	30 Gy	231	2	5
6	28	M	IIA	30 Gy	684	6	5
7	33	F	IIA	30 Gy	260	4	5
8	17	M	IIB	30 Gy	349	6	5
9	34	F	IIA	30 Gy	497	2	5
10	38	M	IIB Bulky	30 Gy	642	2	5

### Volume definition

Target contouring was determined by the pre-chemotherapy imaging (CT and or PET/CT-based). CTV was assumed to be the initial volume of the PET or CT positive lymph-nodes before chemotherapy. Contouring was performed by an experienced radiation oncologist according to GHSG guidelines [23]. PTV was determined adding a three-dimensional 10-mm isotropic margin to the CTV, according to IFRT volume definitions.

### Treatment planning

Treatment planning was performed for all patients with CT scanning with 2-mm slice thickness. Contouring and planning were performed with Varian Eclipse treatment planning system (TPS version 8.6, Varian Systems, Palo Alto, CA**).** Two different immobilization devices were used (5 patients were treated with wing-board and 5 patients with mask). For 3D-CRT planning, a coplanar mono-isocentric single and half-field technique was used for treatment plans, according to target geometry and volume size. Two different beam configurations were used: a pair of parallel-opposed beams (antero-posterior and postero-anterior) in 4 patients, and a multi-fields (4 to 6) irradiation technique with half-field technique in 6 patients. In both cases, a multi leaf collimator was used with a 0.5 cm uniform margin around the PTV.

For all patients, the prescribed dose was 30 Gy to the PTV with a dose homogeneity of 5% and 7%, as recommended by the International Commission on Radiation Units and Measurements Report No. 50 [24]. Treatments were performed with a 6 MV photon beam from Varian Clinac DHX equipped with a 120 leaf Millennium Multileaf collimator.

Treatment plans with IMRT were performed maintaining the same target coverage and applying the same dose scheme of 2 Gy × 15. The actual 3D-CRT plans were retrieved and replanned for the IMRT study using the same treatment planning system. This system uses a gradient-search based inverse planning algorithm to generate optimal beam fluences, for which planners specify the dose objectives/constraints for the target and all other normal structures. The goal of the optimization in the present study was to minimize an objective function as defined on the basis of the difference between the desired and calculated doses for the target and all specified critical organs.

All IMRT plans were designed with five equally spaced beams centered on the target volume (gantry angles at 0°, 72°, 144°, 216°, and 288°). All plans were designed and calculated with sliding window radiation delivery technique.

### Plan comparison

We computed the target coverage as measured by the volume fraction of the target receiving at least 95% of the prescription dose of the PTV (V95), the dose covering at least 95% of the PTV (D95%) and the maximal, minimal and mean PTV dose. The V2, V5, V10, V20 and V30 parameters for the origin of coronary arteries and heart, the V5, V10, V20 and V30 for spinal cord and breast (breast for female patients only) and V5, V10, V20 and V30 for lung, thyroid, oesophagus were calculated for both techniques and then compared. All these parameters were retrieved from differential DVHs.

### NTCP calculation

In order to assess the normal tissue sparing capability of both radiation treatment techniques, normal tissue complication probabilities (NTCPs) for lungs, heart, spinal cord, oesophagus and thyroid were calculated for each patient using a custom MATLAB based code. Only for women, breast NTCP was also evaluated. The dose-volume histogram (DVH) reduction algorithm of Lyman and Wolbarst [25] and the effective volume method introduced by Kutcher et al. [26] were applied to each DVH to produce a value for the NTCP. In this model, the organ dose is described as independent fractional volume elements *v*_*i*_ (such that $\sum_{i}v_{i}=1$), irradiated to doses *d*_*i*_. According to this formalism, the NTCP was calculated as:

(1) $$NTCP=\frac{1}{\sqrt{2\cdot \pi \;}}\int_{-\infty }^{t}\exp\left(-\frac{t^{2}}{2}\right)$$

(2) $$t=\frac{\left(D-TD_{50}(v)\right)}{m\cdot TD_{50}(v)}$$

(3) $$TD_{50}(v)=TD_{50}\cdot v_{\mathrm{eff}}^{-n}$$

(4) $$v_{\mathrm{eff}}={\sum_{i}v_{i}\cdot \left(\frac{d_{i}}{d_{\mathrm{ref}}}\right)}^{\frac{1}{n}}$$

where *TD*_50_ is the tolerance dose for 50% complications for uniform whole organ irradiation and *d*_*ref*_ the reference dose, chosen to be the maximum dose *d*_*max*_. In Eq. 4

(5) $$v_{i}=\left(\frac{V_{i}}{V_{\text{ref}}}\right)$$

where *V*_*ref*_ is the total volume of the organ, *V*_*i*_ the volume fraction, The parameter *m* in Eq. 2 is the slope factor which affects the steepness of the S-shaped dose-response curve, while *n* is the parameter which represents the volume effect. Parameters for NTCP calculations (volume effect *n*, slope *m*, and tolerance doses TD_50_ and TD_5_) were taken from Burman et al. [27] and are shown in Table 2. For each organ at risk (OAR), the different end point is also reported. Given the low doses received by the critical organs, we calculated NTCP corresponding to TD_5/5_ (tolerance doses leading to 5% complication rates at 5 years), with the exception of lung and breast where the TD_50/5_ (tolerance doses leading to 50% complication rates) was calculated.

**Table 2:** Parameters for NTCP calculation

**Organ**	**Size Factor (n)**	**Slope (m)**	**TD**_**5/5**_	**TD**_**50/5**_	**End Point**
Lung	0.87	0.18		24.5	Pneumonities
Heart	0.35	0.10	40		Pericardities
Spinal cord	0.05	0.175	47		Myelities/necrosis
Thyroid	0.22	0.26	45		Thyroidities
Oesophagus	0.06	0.11	55		Clinical stricture/perforation
Breast	0.78	0.27		62.5	Fibrosis

Abbreviations: TD_5/5_ = tolerance doses leading to 5% complication rates at 5 years, TD_50/5_ = tolerance doses leading to 50% complication rates at 5 years.

### Statistical analysis

Analyses were performed using a paired two-tailed Student *t* test to determine if there was a significant difference between all the parameters obtained with 3D-CRT and IMRT. Differences were considered statistically significant at p ≤ 0.05. Statistical analysis was performed with Origin Pro 8.5 package (OriginLab Corporation, Northampton USA).

## Results

All ten patients completed their prescribed therapeutic schedule. The radiation treatment started at a median of 50 days (range 31-73) from the end of chemotherapy. During radiation therapy, no acute toxicity was recorded in 5 patients, while 4 patients presented dysphagia G1-G2 and 1 patient presented dysphagia G1 and erythema G1. All patients were in complete response at the restanging after radiation therapy and no relapses were recorded after a median follow-up of 34 months (range 24-48). No late toxicities were recorded.

### PTV coverage

**Figure 1:**
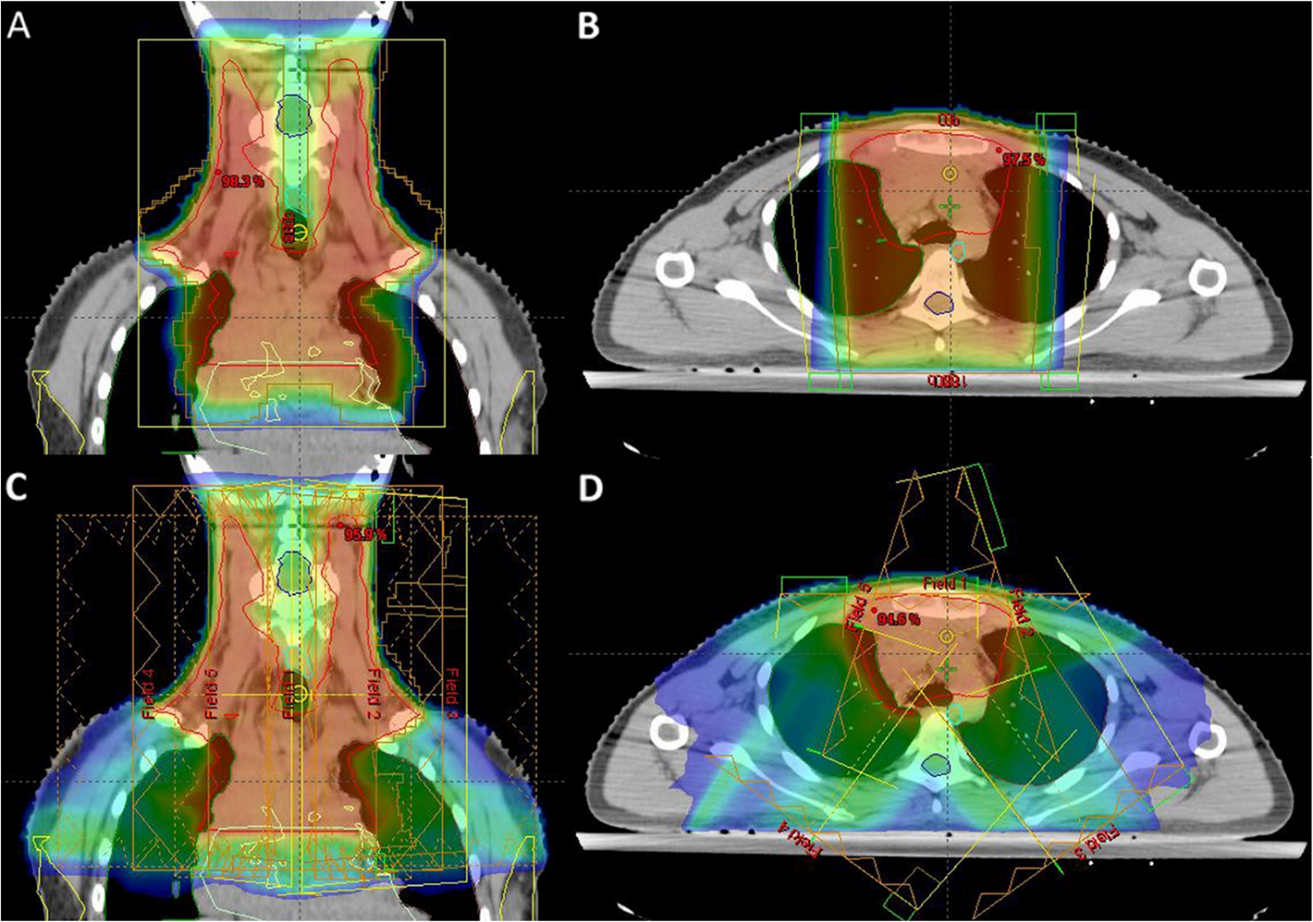
Comparison of dose distribution and PTV (red line) coverage of 3D-CRT (A,B) vs IMRT (C,D) plans in coronal (A,C) and axial (B,D) section.

As shown in Table 3, the D95 was better with IMRT respect to 3D-CRT with statistically significant differences (p = 0.028) while no statistically significant difference was recorded for V95 between the two techniques. For all patients, the mean dose to the target volume was similar for both treatments. The minimum dose was significantly higher for IMRT, while the maximum dose and the median dose were significantly higher for 3D-CRT (Figure 1).

**Table 3:** Comparison between PTV dose parameters of 3D-CRT and IMRT plans

**Parameter**	**3D-CRT**	**IMRT**	**p-value**
Dose minimum (%)	41.4 (23.3 - 65.2)	71.4 (31.2 – 87.7)	<0.001
Dose maximum (%)	108.8 (105.2 – 112.6)	106.6 (104.2 – 108.7)	0.030
Dose mean (%)	100.7 (98.2 – 103.8)	100.0 (99.5 – 100.3)	0.294
Dose median (%)	101.4 (99.4 – 103.8)	100.1 (99.5 – 100.4)	0.049
V95 (%)	29.2 (13.9 - 47.0)	27.7 (0.02 – 75.7)	0.619
D95 (%)	27.7 (25.2 – 29.5)	28.8 (28.2 - 29.2)	0.028

### Dose to thyroid

The mean thyroid dose was 15.25 Gy with 3D technique compared to 21.4 Gy with IMRT, with a statistically significant difference (p = 0.02). The minimum dose was 2.74 Gy with 3D technique compared to 10.7 Gy with IMRT (p = 0.003) whereas the maximum dose was 27.9 Gy with 3D technique and 30.8 Gy with IMRT.

As shown in Table 4, the mean V10 parameter was 64.8 for 3D-CRT and 95 for IMRT (p = 0.0019) while the other volumetric parameters (V20 and V30) showed no statistically significant difference between 3D-CRT and IMRT.

**Table 4:** Dose-volume parameters for OAR

	***3D-CRT***	***IMRT***	***p-value***
**Thyroid**			
V10	64.8	95	0.0019
V20	46.4	54.6	0.48
V30	16.2	9.6	0.28
**Left Lung**			
V5	28.9	48.1	0.001
V10	20.5	30.0	0.13
V20	13.2	7.8	0.21
V30	4.7	0.1	0.014
**Right Lung**			
V5	30.3	44.8	0.03
V10	23.2	32.8	0.17
V20	14.7	7.9	0.19
V30	2.59	0.1	0.017
**Esophagus**			
V10	49.7	54.6	0.48
V20	31.1	33.7	0.35
V30	6.42	0.33	0.02
**Spinal Cord**			
V5	56.7	66.2	0.25
V10	41.4	55.9	0.07
V20	17.8	7.2	0.02
V30	4.7	0	0.07
**Heart**			
V2	20	26	0.55
V5	15.8	25.7	0.63
V10	12.9	15.3	0.76
V20	7.6	6.5	0.79
V30	1.4	0.2	0.24
**Coronary arteries**			
V2	50.7	62	0.46
V5	44	52.8	0.57
V10	39.2	44.5	0.72
V20	25.7	23	0.78
V30	6.2	0.94	0.10
**Left breast**			
V5	4.14	20.6	0.018
V10	2.5	13.6	0.035
V20	1.08	0	0.09
V30	0.15	0	0.34
**Right breast**			
V5	3.3	17	0.059
V10	1.7	11	0.07
V20	0.9	0	0.04
V30	0.82	0	0.34

Despite the higher mean dose delivered to the thyroid gland by IMRT, no statistically significant differences were found between NTCP values (p = 0.56) (Table 5).

**Table 5:** NTCP for OAR

**OAR**	**3D-CRT**	**IMRT**	**p-value**
Thyroid	2.98 (0.01 - 6)	3.35 (0.01 - 5.5)	0.56
Lungs	0.2 (0.02 - 0.60)	0.08 (0.02 - 0.40)	0.033
Spinal Cord	0.70 (0.01 - 1.40)	0.17 (0.01 - 1.20)	0.004
Breast	0.6 (0.0.1 - 1.5)	0.82 (0.01 - 1.4)	0.086
Oesophagus	<0.0001	<0.0001	-
Heart	<0.0001	<0.0001	-
Coronary arteries	<0.0001	<0.0001	-

Differences were considered statistically significant at p ≤ 0.05.

### Dose to oesophagus

The mean oesophagus dose was 14.17 Gy with 3D technique compared to 12.93 Gy with IMRT, while the minimum received dose was 0.84 Gy with 3D technique and 1.67 Gy with IMRT. Maximum doses were 30.52 Gy and 30.2 for 3D and IMRT, respectively. The mean V30 parameter was 6.42 for 3D-CRT and 0.33 for IMRT (p = 0.02) while the other volumetric parameters showed no statistically significant difference between 3D-CRT and IMRT.

Both for 3D and for IMRT treatment NTCP resulted negligible for all patients.

### Dose to spinal cord

The mean spinal cord dose was 11.18 Gy with 3D technique compared to 9.18 Gy with IMRT, the lowest dose was 0.21 Gy with 3D technique compared to 0.31 Gy with IMRT; the maximum dose was 30.2 Gy 3D technique compared to 19.96 Gy with IMRT (p <0.001).

The mean V20 parameter was 17.8 for 3D-CRT and 7.2 for IMRT (p = 0.02) while the other volumetric parameters showed no statistically significant difference between 3D-CRT and IMRT.

For each patient, the NTCP calculated for spinal cord resulted statistically significant higher for 3D than for IMRT treatment (p = 0.004).

### Dose to heart and coronaries arteries

For the heart, the average dose was equal to 4.25 Gy with 3D technique compared to 4.21 Gy with IMRT, the lowest dose was 0.25 Gy with 3D technique compared to 0.26 Gy with IMRT while the highest dose was equal to 24.3 Gy with 3D technique compared with 21.94 Gy with IMRT, without statistical differences. In particular, for emergency coronary artery the mean dose was equal to 9.94 Gy with 3D technique compared to 8.98 Gy with IMRT (p = 0.77). The statistical comparison of V2, V5, V10, V20 and V30 between 3D-CRT and IMRT showed no difference.

Both for 3D and IMRT treatment NTCP resulted negligible for all patients.

### Dose to lung

Although the mean lung dose was equal to 6.75 Gy with 3D technique compared to 8.69 Gy with IMRT, no statistically significant difference was recorded between these two treatments. The lowest dose was 0.13 Gy with 3D-CRT compared to 0.14 Gy with IMRT whereas the maximum dose was 31.7 Gy with 3D technique compared to 30.7 Gy with IMRT, without statistically significant differences.

Regarding the volumetric parameters, for the left lung the V5 resulted 28.9 in 3D-CRT group respect to V5 in IMRT group that resulted 48.1 (p = 0.001), while for the right lung V5 was 30.3 in 3D-CRT and 44.8 in IMRT (p = 0.03). Considering the V30, for the left lung it was 0.1 with IMRT respect to 3D-CRT that resulted 4.7 (p = 0.014) and for the right lung the V30 was 0.1 with IMRT respect to V30 of 2.59 with 3D-CRT (p = 0.017). No statistically significant differences were recorded for V10 and V20 parameters.

NTCP values showed no statistically significant differences, with p = 0.081.

### Dose to breast

The mean dose to the breast was 0.97 Gy with 3D technique compared to 2.32 Gy with IMRT, with a statistically significant difference (p <0.0017). The lowest dose was 0.06 with 3D technique compared to 0.3 Gy with IMRT and the maximum dose was 28.6 Gy with 3D technique compared to 20.29 Gy with IMRT (p <0.0001).

Regarding the volumetric parameters for the left breast the mean V5 parameter was 4.14 for 3D-CRT and 20.6 for IMRT (p = 0.018) while the mean V10 was 2.5 for 3D-CRT and 13.6 for IMRT (p = 0.035). The same parameters for the right breast was as following: a mean V5 of 3.3 for 3D-CRT and 17 for IMRT (p = 0.059) and a mean V10 of 1.7 for 3D-CRT and 11 for IMRT (p = 0.07). No statistically significant differences were recorded for V20 and V30 parameters for left and right breast.

NTCP resulted comparable both for left and right breast with p = 0.18 and p = 0.086, respectively.

## Discussion

The main question addressed in our study is whether IFRT IMRT is a suitable technique for the treatment of supra-diaphragmatic early stage HL patients and if it can replace IFRT 3DCRT. In our study the PTV coverage obtained with IMRT was significantly better compared with 3D-CRT and it depends on IMRT ability to modulate the intensity of each radiation beam that resulting on a high conformal delivery of total dose radiation to PTV. Our results were similar to those reported by other authors, who showed that IMRT treatment was superior to 3DCRT plans regarding the PTV coverage [13-17]. This physical and dosimetric concept can be translated in a maximization of the actual total delivered dose to the target volume and therefore with an increased probability of local control.

The open question is whether IMRT might lead to a lower dose to the surrounding organs and whether the rate of late complication could be reduced with IMRT, in particular lung toxicity and cardiac toxicity. In our study, IMRT showed an advantage respect to the 3D-CRT technique for the oesophagus and spinal cord. This dose reduction could translate in a less acute and late morbidity. In particular, the NTCP, V20 and maximum dose for spinal cord is significantly lower for IMRT than for 3D treatment in every patient. In case of mediastinal relapse, it could be important to reduce the total dose delivered to the spinal cord for eventual re-irradiation. About thyroid, in our study the dose parameters analysis showed that IMRT is no advantageous respect to 3D-CRT. When supraclavear and/or neck lymph nodes were included in the treatment volume, the multi-fields IMRT arrangement was not able to spare the thyroid glands. On the contrary, when a 3D-CRT (Antero-Posterior and Postero-Anterior in particular) was employed, the most part of thyroid gland was not included in the treatment fields. The mean actual dose delivered with 3DCRT to thyroid gland in our patients was 15.25 Gy, statistically lower than the 21.4 Gy with IMRT. When we considered the volumetric parameter V10, we showed also a statistically significant advantage for 3D-CRT respect to IMRT technique. No difference was recorded respect to NTCP parameters. Compared with the general population, long-term survivors of Hodgkin’s lymphoma have a higher incidence of thyroid abnormalities, including hypothyroidism, hyperthyroidism and thyroid neoplasms [28]. Thyroid cancer is the second most common tumor reported among HL survivor patients. The Late Effect Study Group reported a 36-fold increased risk of thyroid cancer, with 95% of these cancers developing within the radiation field [29]. In 461 paediatric HL patients, the 20-year estimated cumulative incidence of hypothyroidism stratified for radiation dose was 30% in patients who received 21 Gy or less and 61% in patients who received more than 21 Gy [30]. Thus, it is reasonable that lowering the radiation dose to thyroid gland could reduce the risk of late toxicity as hypothyroidism and second cancer. We reported no difference between 3DCRT and IMRT regarding the coronary arteries and heart dose parameters evaluation, as well as for the NTCP. Although no difference in the mean dose was reported by Girinsky et al., they showed a reduction of V30 with IMRT plan. Other authors stated that the aim of heart sparing was best achieved with IMRT although complete elimination of high dose to some parts of the heart was not possible with any technique [13,14,18]. Therefore, moving from conventional radiation techniques to IMRT would reduce the risk of cardiac morbidity and mortality but the individual magnitude of clinical benefit is hard to predict. Moreover the patient anatomy, comorbidity and pre-existent hearth damage could be important adjunctive risk factors. One of the objectives of using IMRT for patients with HL is to reduce the radiation dose to the surrounding lung. No agreement was reached regarding a correlation between lung toxicity and IMRT in HD patients. Although a reduction of the mean dose to the lung with IMRT plans was reported, on the other hand an increase of V20 using IMRT planning was recorded in other papers [13,14,17]. We showed a reduction of V30 and of NTCP value using IMRT that reach a statistical significance while the V5 parameter was increased with IMRT. The relationship between this dose-volume constraint and the risk of lung toxicity has not been clearly defined. Moreover, whether low dose to a large volume is more important than higher dose to a smaller volume in the development of pneumonitis is unknown. In this context, the role of IMRT in supra-diaphragmatic HL patients remains largely unknown owing the concern that IMRT may deliver a low, yet damaging dose to normal lung tissue.

Finally, literature data seem uniformly believe that IMRT technique increases the dose delivered to the breasts in female patients with HL [13-15,31]. Also in our experience, the 3D-CRT offers a more convenient dose distribution to the breast, in particular to the left breast, respect to IMRT. The impact of the low dose radiation in a large treated volume of the breasts related to the IMRT technique should be investigated in a prospective setting [15,31]. The late occurrence of second breast cancer in HL survivors is a topic of paramount importance. In this cohort of patients cumulative absolute risks of breast cancer after 10, 20 and 30 years after the radiation treatment were 1.4%, 11.1% and 29.0%, respectively [7]. Dosimetric studies showed IMRT as superior to 3D-CRT in terms of target coverage, conformity, and sparing of normal tissue but concerns has been raised about its carcinogenic risk [31-34]. It has been estimated that IMRT may increase the risk of second cancer by a factor of 1.2-8 but we must point out that now we have only dosimetric studies and no clinical data, given the short follow-up of patients treated with IMRT so far [28-30]. Other issues that should be considered are the time and resources required for IMRT planning. In fact we acknowledge that IMRT is quite a time consuming and labor-intensive procedure if compared to 3D-CRT (increased time for planning, delineation, dose delivery and quality assurance).

In our opinion, IMRT can be used in patients with HL supra-diaphragmatic, showing itself to be superior to 3D-CRT in terms of dose distribution within the target and reduction of dose to organs at risk, especially lung and bone marrow. It is worth noting that in the present study the number of fields used in 3D-CRT planning were sometimes different. However we believe that this could have a minimum impact on the target coverage (since similar average doses were obtained), even if it might play a role on the dose delivered to the OARs. Regarding the dose delivered to thyroid with IMRT technique, the decision should be made on a case by case basis. We point out also that in the absence of clinical data, IMRT should be used with caution in HL women patients. In conclusion, IMRT is a seducing treatment option in patients with early stage HL, but a number of theoretical and practical hurdles remain to be resolved before it can be used routinely in clinical daily practice

## Competing interests

The authors declare that they have no competing interests.

## Authors’ contributions

VDS and MDA participated in design and coordination of study. VDS, CB, MDA, SB, AF and MCC participated in data acquisition. VDS, MDA and SB contributed to the statistical analysis. VDS, MDA, CB, SB, MV, MFO and GM contributed to interpretation of data. VDS and MDA drafted the article. LC and RME critically reviewed/revised the article. All authors read and approved the final manuscript.

## References

[B1] Castellino SM Geiger AM Mertens AC Leisenring WM Tooze JA Goodman P Stovall M Robison LL Hudson MM Morbidity and mortality in long-term survivors of Hodgkin lymphoma: a report from the Childhood Cancer Survivor Study Blood 2011 117 1806 1816 10.1182/blood-2010-04-278796 21037086 PMC3056636

[B2] Sklar C Whitton J Mertens A Stovall M Green D Marina N Greffe B Wolden S Robison L Abnormalities of the thyroid in survivors of Hodgkin's disease: data from the Childhood Cancer Survivor Study J Clin Endocrinol Metab 2000 85 3227 3232 10.1210/jc.85.9.3227 10999813

[B3] Bhatia S Ramsay NK Bantle JP Mertens A Robison LL Thyroid Abnormalities after Therapy for Hodgkin's Disease in Childhood Oncologist 1996 1 62 67 10387970

[B4] Milano MT Constine LS Okunieff P Normal tissue tolerance dose metrics for radiation therapy of major organs Semin Radiat Oncol 2007 17 131 140 10.1016/j.semradonc.2006.11.009 17395043

[B5] Yorke ED Jackson A Rosenzweig KE Merrick SA Gabrys D Venkatraman ES Burman CM Leibel SA Ling CC Dose-volume factors contributing to the incidence of radiation pneumonitis in non-small-cell lung cancer patients treated with three-dimensional conformal radiation therapy Int J Radiat Oncol Biol Phys 2002 54 329 339 12243805 10.1016/s0360-3016(02)02929-2

[B6] Hudson MM Poquette CA Lee J Greenwald CA Shah A Luo X Thompson EI Wilimas JA Kun LE Crist WM Increased mortality after successful treatment for Hodgkin's disease J Clin Oncol 1998 16 3592 3600 9817280 10.1200/JCO.1998.16.11.3592

[B7] Travis LB Hill D Dores GM Gospodarowicz M van Leeuwen FE Holowaty E Glimelius B Andersson M Pukkala E Lynch CF Pee D Smith SA Van’t Veer MB Joensuu T Storm H Stovall M Jr Boice JD Gilbert E Gail MH Cumulative absolute breast cancer risk for young women treated for Hodgkin Lymhoma J Natl Cancer Inst 2005 97 1428 1437 10.1093/jnci/dji290 16204692

[B8] Van Leeuwen FE Klokman WJ Stovall M Dahler EC van’t Veer MB Noordijk EM Crommelin MA Aleman BM Broeks A Gospodarowicz M Travis LB Russell NS Roles of radiation dose, chemotherapy, and hormonal factors in breast cancer following Hodgkin's disease J Natl Cancer Inst 2003 95 971 980 10.1093/jnci/95.13.971 12837833

[B9] Hodgson DC Gilbert ES Dores GM Schonfeld SJ Lynch CF Storm H Hall P Langmark F Pukkala E Andersson M Kaijser M Joensuu H Fosså SD Travis LB Long-term solid cancer risk among 5-year survivors of Hodgkin's lymphoma J Clin Oncol 2007 25 1489 1497 10.1200/JCO.2006.09.0936 17372278

[B10] Engert A Plütschow A Eich HT Lohri A Dörken B Borchmann P Berger B Greil R Willborn KC Wilhelm M Debus J Eble MJ Sökler M Ho A Rank A Ganser A Trümper L Bokemeyer C Kirchner H Schubert J Král Z Fuchs M Müller-Hermelink HK Müller RP Diehl V Reduced treatment intensity in patients with early-stage Hodgkin's lymphoma N Engl J Med 2010 363 640 652 10.1056/NEJMoa1000067 20818855

[B11] Girinsky T van der Maazen R Specht L Aleman B Poortmans P Lievens Y Meijnders P Ghalibafian M Meerwaldt J Noordijk E Involved-node radiotherapy (INRT) in patients with early Hodgkin lymphoma: concepts and guidelines Radiother Oncol 2006 79 270 277 10.1016/j.radonc.2006.05.015 16797755

[B12] Kirova YM Chargari C Applications of new irradiation modalities in patients with lymphoma: Promises and uncertainties World J Radiol 2011 3 66 69 10.4329/wjr.v3.i3.66 21512653 PMC3080052

[B13] Goodman KA Toner S Hunt M Wu EJ Yahalom J Intensity-modulated radiotherapy for lymphoma involving the mediastinum Int J Radiat Oncol Biol Phys 2005 62 198 206 10.1016/j.ijrobp.2004.08.048 15850922

[B14] Nieder C Schill S Kneschaurek P Molls M Comparison of the three different mediastinal radiotherapy techniques in female patients: impact on heart sparing and dose to the breast Radiother Oncol 2007 82 301 307 10.1016/j.radonc.2006.10.015 17156873

[B15] Weber DC Peguret N Dipasquale G Cozzi L Involved-node and involved-field volumetric modulated arc vs fixed beam intensity- modulated radiotherapy for female patients with early stage supra-diaphragmatic hodgkin lymphoma: a comparative planning study Int J Radiat Oncol Biol Phys 2009 75 1578 1586 10.1016/j.ijrobp.2009.05.012 19596171

[B16] Chera BS Rodriguez C Morris CG Louis D Yeung D Li Z Mendenhall NP Dosimetric comparison of three different involved nodal irradiation techniques for stage II hodgkin’s lymphoma patients: conventional radiotehrapy, intensity-modulated radiotehrapy and three-dimensional proton radiotherapy Int J Radiat Oncol Biol Phys 2009 75 1173 1180 10.1016/j.ijrobp.2008.12.048 19386423

[B17] Girinsky T Pichenot C Beaudre A Ghalibafian M Lefkopoulos D Is intensity-modulated radiotherapy better than conventional radiation treatment and three-dimensional conformal radiotherapy for mediastinal masses in patients with hodgkin’s disease, and is there a role for beam orientation optimization and dose constraints assigned to virtual volumes? Int J Radiat Oncol Biol Phys 2006 64 218 226 10.1016/j.ijrobp.2005.06.004 16169675

[B18] Cella L Liuzzi R Magliulo M Conson M Camera L Salvatore M Pacelli R Radiotherapy of large target volume in Hodgkin’s lymphoma: normal tissue sparing capability of forward IMRT versus conventional techniques Radiation Oncol 2010 11 5 33 10.1186/1748-717X-5-33 PMC2881006 20459790

[B19] Koeck J Abo-Madyan Y Lohr F Stieler F Kriz J Mueller RP Wenz F Eich HT Radiotherapy for early mediastinal Hodgkin lymphoma according to the German Hodgkin Study Group (GHSG): the roles of intensity-modulated radiotherapy and involved-node radiotherapy Int J Radiat Oncol Biol Phys 2012 83 268 276 10.1016/j.ijrobp.2011.05.054 22079733

[B20] Sripada PV Tenali SG Vasudevan M Viswanadhan S Sriraman D Kandasamy R Hybrid (COPP/ABV) therapy in childhood Hodgkin's disease: a study of 53 cases during 1989-1993 at the Cancer Institute, Madras Paediatric Hematol Oncol 1995 4 333 341 10.3109/08880019509029583 7577385

[B21] Santoro A Bonadonna G Valagussa P Zucali R Viviani S Villani F Pagnoni AM Bonfante V Musumeci R Crippa F Long-term results of combined chemotherapy-radiotherapy approach in Hodgkin's disease: superiority of ABVD plus radiotherapy versus MOPP plus radiotherapy J Clin Oncol 1987 5 27 37 2433409 10.1200/JCO.1987.5.1.27

[B22] Cox JD Stetz J Pajak TF Toxicity criteria of the Radiation Therapy Oncology Group (RTOG) and the European Organization for Research and Treatment of Cancer (EORTC) Int J Radiat Oncol Biol Phys 1995 31 1341 1346 10.1016/0360-3016(95)00060-C 7713792

[B23] Eich HT Engenhart-Cabillic R Hansemann K Lukas P Schneeweiss A Seegenschmiedt H Skripnitchenko R Staar S Willich N Muller RP Quality control of involved field radiotherapy in patients with early-favorable (HD10) and early-unfavorable (HD11)hodgkin's lymphoma: an analysis of the German Hodgkin Study Group Int J Radiat Oncol Biol Phys 2008 71 1419 1424 10.1016/j.ijrobp.2007.12.002 18234433

[B24] International Commission on Radiation Units and Measurements Report no. 50. Prescribing, recording, and reporting photon beam therapy 1993 ICRU, Washington

[B25] Lyman JT Wolbarst AB Optimization of radiation therapy, III: a method of assessing complications probabilities from dose-volume histograms Int J Radiat Oncol Biol Phys 1987 13 103 109 3804804 10.1016/0360-3016(87)90266-5

[B26] Kutcher GJ Burman C Calculation of complication probability factors for non-uniform normal tissue irradaiation: the effective volume method Int J Radiat Oncol Biol Phys 1989 16 1623 1630 10.1016/0360-3016(89)90972-3 2722599

[B27] Burman C Kutcher GJ Emami B Goiten M Fitting of normal tissue tolerance data to an analytic function Int J Radiat Oncol Biol Phys 1991 21 123 135 2032883 10.1016/0360-3016(91)90172-z

[B28] Chronowski GM Wilder RB Tucker SL Ha CS Younes A Fayad L Rodriguez MA Hagemeister FB Barista I Cabanillas F Cox JD Analysis of in-field control and late toxicity for adults with early stage Hodgkin’s disease treated with chemotherapy followed by radiotherapy Int J Radiat Oncol Biol Phys 2003 55 36 43 10.1016/S0360-3016(02)03915-9 12504034

[B29] Bhatia S Yasui Y Robison LL Birch JM Bogue MK Diller L DeLaat C Fossati-Bellani F Morgan E Oberlin O Reaman G Ruymann FB Tersak J Meadows AT Late Effects Study Group High risk of subsequent neoplasms continues with extended follow-up of childhood Hodgkin’s disease: report from the late effects study group J Clin Oncol 2003 21 4386 4394 10.1200/JCO.2003.11.059 14645429

[B30] Metzger ML Hudson MM Somes GW Shorr RI Li CS Krasin MJ Shelso J Pui CH Howard SC White race as a risk factor for hypothyroidism after treatment for pediatric Hodgkin’s lymphoma J Clin Oncol 2006 24 1516 1521 10.1200/JCO.2005.05.0195 16575001

[B31] Weber DC Johanson S Peguret N Cozzi L Olsen DR Predicted risk of radiation-induced cancers after involved field and involved node radiotherapy with or without intensity modulation for early-stage Hodgkin lymphoma in female patients Int J Radiat Oncol Biol Phys 2011 81 490 497 10.1016/j.ijrobp.2010.05.035 20800383

[B32] Hall EJ Wuu CS Radiation-induced second cancers: the impact of 3D-CRT and IMRT Int J Radiat Oncol Biol Phys 2003 56 83 88 10.1016/S0360-3016(03)00073-7 12694826

[B33] Hall EJ Intensity modulated radiation therapy, protons, and the risk of second cancer Int J Radiat Oncol Biol Phys 2006 65 1 7 10.1016/j.ijrobp.2006.01.027 16618572

[B34] Kry SF Salehpour M Followill DS Stovall M Kuban DA White RA Rosen II The calculated risk of fatal secondary malignancies from intensity-modulated radiation therapy Int J Radiat Oncol Biol Phys 2005 62 1195 1203 10.1016/j.ijrobp.2005.03.053 15990025

